# High incidence of PTSD diagnosis and trauma-related symptoms in a trauma exposed bipolar I and II sample

**DOI:** 10.3389/fpsyt.2022.931374

**Published:** 2022-10-20

**Authors:** Bridget Hogg, Alicia Valiente-Gómez, Diego Redolar-Ripoll, Itxaso Gardoki-Souto, Marta Fontana-McNally, Walter Lupo, Esther Jiménez, Mercè Madre, Laura Blanco-Presas, María Reinares, Romina Cortizo, Anna Massó-Rodriguez, Juan Castaño, Isabel Argila, José Ignacio Castro-Rodríguez, Mercè Comes, Marta Doñate, Elvira Herrería, Cristina Macias, Estanislao Mur, Patricia Novo, Adriane R. Rosa, Eduard Vieta, Joaquim Radua, Frank Padberg, Victor Pérez-Solà, Ana Moreno-Alcázar, Benedikt L. Amann

**Affiliations:** ^1^Centre Fòrum Research Unit, Institute of Neuropsychiatry and Addiction (INAD), Parc de Salut Mar, Barcelona, Spain; ^2^Hospital del Mar Medical Research Institute (IMIM), Barcelona, Spain; ^3^PhD Programme, Department of Psychiatry and Forensic Medicine, Universitat Autònoma de Barcelona, Barcelona, Spain; ^4^Centro de Investigación Biomédica en Red de Salud Mental (CIBERSAM), Instituto Carlos III, Madrid, Spain; ^5^Neuromodulation Unit, Institut Brain, Barcelona, Spain; ^6^Cognitive NeuroLab, Faculty of Psychology and Educational Sciences, Universitat Oberta de Catalunya (UOC), Barcelona, Spain; ^7^Bipolar and Depressive Disorders Unit, Hospital Clinic, University of Barcelona, L'Institut d'Investigacions Biomèdiques August Pi i Sunyer (IDIBAPS), Barcelona, Spain; ^8^Addictive Behaviours Unit, Psychiatry Department, Hospital de la Santa Creu i Sant Pau, Barcelona, Spain; ^9^Hospital Benito Menni-CASM, Sant Boi de Llobregat, Barcelona, Spain; ^10^FIDMAG Germanes Hospitalàries Research Foundation, Barcelona, Spain; ^11^Programa TEPT-AGRESX, Instituto de Neurociencias (ICN), Hospital Clinic, Barcelona, Spain; ^12^Centro Salud Mental Adultos Ciutat Vella, Parc Sanitari Sant Joan de Déu, Barcelona, Spain; ^13^Centro Salud Mental Adultos (CSMA), Institute of Neuropsychiatry and Addictions (INAD), Parc de Salut Mar, Barcelona, Spain; ^14^Centro de Salud Mental Infantil y Juvenil (CSMIJ), Institute of Neuropsychiatry and Addictions (INAD), Parc de Salut Mar, Barcelona, Spain; ^15^Parc Sanitari Sant Joan de Deu, Sant Boi de Llobegrat, Spain; ^16^Centre Emili Mira, Institute of Neuropsychiatry and Addictions (INAD), Parc de Salut Mar, Barcelona, Spain; ^17^Day Hospital, Centro de Psicoterapia de Barcelona (CPB), Barcelona, Spain; ^18^Laboratory of Molecular Psychiatry, Hospital de Clínicas de Porto Alegre (HCPA), Porto Alegre, Brazil; ^19^Departamento de Farmacologia, Instituto de Ciéncias Básicas de Saúde, Universidade Federal do Rio Grande do Sul, Porto Alegre, Brazil; ^20^Postgraduate Program in Psychiatry and Behavioral Sciences, Federal University of Rio Grande doSul (UFRGS), Porto Alegre, Brazil; ^21^Department of Clinical Neuroscience, Karolinska Institutet (KI), Solna, Sweden; ^22^Department of Psychosis Studies, Institute of Psychiatry, Psychology and Neuroscience, King's College London, London, United Kingdom; ^23^Clinic for Psychiatry and Psychotherapy, Klinikum der Universität München, Munich, Germany; ^24^Departamento de Medicina y Ciencias de la Vida, Universitat Pompeu Fabra, Barcelona, Spain

**Keywords:** bipolar disorder, PTSD—post-traumatic stress disorder, psychological trauma, sexual abuse, physical abuse and neglect, dissociation

## Abstract

**Background:**

Post-traumatic stress disorder (PTSD) is an established comorbidity in Bipolar Disorder (BD), but little is known about the characteristics of psychological trauma beyond a PTSD diagnosis and differences in trauma symptoms between BD-I and BD-II.

**Objective:**

(1) To present characteristics of a trauma-exposed BD sample; (2) to investigate prevalence and trauma symptom profile across BD-I and BD-II; (3) to assess the impact of a lifetime PTSD diagnosis vs. a history of trauma on BD course; and (4) to research the impacts of sexual and physical abuse.

**Methods:**

This multi-center study comprised 79 adult participants with BD with a history of psychological trauma and reports baseline data from a trial registered in Clinical Trials (https://clinicaltrials.gov; ref: NCT02634372). Clinical variables were gathered through clinical interview, validated scales and a review of case notes.

**Results:**

The majority (80.8%) of our sample had experienced a relevant stressful life event prior to onset of BD, over half of our sample 51.9% had a lifetime diagnosis of PTSD according to the Clinician Administered PTSD scale. The mean Impact of Event Scale-Revised scores indicated high levels of trauma-related distress across the sample, including clinical symptoms in the PTSD group and subsyndromal symptoms in the non-PTSD group. Levels of dissociation were not higher than normative values for BD. A PTSD diagnosis (vs. a history of trauma) was associated with psychotic symptoms [2(1) = 5.404, *p* = 0.02] but not with other indicators of BD clinical severity. There was no significant difference between BD-I and BD-II in terms of lifetime PTSD diagnosis or trauma symptom profile. Sexual abuse significantly predicted rapid cycling [2(1) = 4.15, *p* = 0.042], while physical abuse was not significantly associated with any clinical indicator of severity.

**Conclusion:**

Trauma load in BD is marked with a lack of difference in trauma profile between BD-I and BD-II. Although PTSD and sexual abuse may have a negative impact on BD course, in many indicators of BD severity there is no significant difference between PTSD and subsyndromal trauma symptoms. Our results support further research to clarify the role of subsyndromic PTSD symptoms, and highlight the importance of screening for trauma in BD patients.

## Introduction

Bipolar disorder (BD) is a severe mental illness which negatively impacts life expectancy (1) and is characterized by depressive and at least one manic or hypomanic episode in the case of BD Type I (BD-I), and by depressive and at least one hypomanic episode in the case of BD Type 2 (BD-II) (2). The aetiology of BD is understood to be complex, involving multiple genes (3) and *gene x environment* interactions (4), as well as environmental risk factors (5).

One factor which has received increasing attention in the aetiology and prognosis of BD is psychological trauma, which predicts increased comorbidity with other mental and somatic disorders (6), including, notably, post-traumatic stress disorder (PTSD) (7). The prevalence of PTSD in bipolar patients has been estimated between 4 and 40% according to reviews (8, 9), compared to an estimated lifetime prevalence of 6.2% in the general population (10). Research suggests there may be a higher prevalence of PTSD in patients with BD-I compared to BD-II (9, 11), but the symptom presentation appears to be similar across both subtypes (11).

Comorbidity of PTSD and BD leads to a higher symptom burden and lower quality of life (9), and psychological trauma during childhood has been associated with a more severe form of the disease: it has been implicated in an earlier onset of BD, increased suicidality, increased substance abuse, lower functioning, more hospitalizations, and faster cycling frequencies (6, 7, 12–14). It has also been associated with more psychosocial stressors occurring before the first and most recent affective episodes (6). Psychological trauma may partially mediate the relationship between a family history of mood disorder and its expression (15), and studies have shown that early life stress may interact with genes of several biological pathways to lead to a poorer prognosis in BD, including lower age at onset, or increased suicide risk (4, 12). Even at the subsyndromal level, post-traumatic stress symptoms are, like full PTSD, associated with significant social and work impairment and a greater number of suicide attempts in the general population (16), and were found to be associated with increased symptoms of anxiety in BD patients during the COVID-19 pandemic (17).

Evidence suggests that different forms of psychological trauma can increase the risk of psychiatric disorders in different ways (18). Physical, emotional, and sexual abuse in childhood are all independently associated with a greater risk of BD (15), and have all been shown to predict an increased number of suicide attempts and lower age of onset (6, 14, 19, 20), as well as cognitive impairment (21). However, childhood physical abuse and sexual abuse have also both been found to be associated with faster cycling frequencies, more substance abuse and comorbidity with other disorders, and more psychosocial stressors occurring before the first and most recent affective episode (6, 19, 20). Specifically, sexual abuse has been shown to be the strongest predictor of rapid cycling (20), and also to be associated with an increased number of mood episodes and with psychotic episodes (14). Meanwhile, physical abuse was associated with self-harm episodes, and both emotional abuse and physical abuse were associated with lower functioning (14), while more research is needed into the impact of emotional abuse in severe mental illness (22).

Despite strong evidence of the link between trauma and BD, and high comorbidity with PTSD, there has been little focus on how to treat this comorbidity (9). Additionally, more research is needed into dissociative disorders in patients with BD and other severe mental illnesses (22). To address these gaps in current research, a multi-center study (23) was implemented to evaluate the effectiveness of a trauma-focused psychotherapy called Eye Movement Desensitization and Reprocessing (EMDR) (24) in a trauma-exposed sample of adult BD I and BD II patients. During the baseline visit for this trial, we collected data covering the detailed retrospective trauma history, symptoms related to reported trauma and dissociation, and the clinical characteristics of a sample of 79 participants with BD and a history of psychological trauma.

In this paper, we present this data, the first to our knowledge to review the sociodemographic and clinical characteristics of a trauma-exposed bipolar disorder sample, with or without the presence of a diagnosis of PTSD. Based on the aforementioned research, we hypothesized that there would be significantly greater comorbidity between BD-I and PTSD and BD-II and PTSD, but that there would not be significant differences in the presentation of trauma symptoms according to BD subtype. Furthermore, we hypothesized that comorbidity with PTSD in a sample of traumatized BD patients would be associated with a worse disease course, and that reported sexual abuse and physical abuse would each be associated with a worse disease course. Therefore, our primary research objectives were the following:

1. To present the sociodemographic, trauma, and clinical characteristics of a sample of trauma-exposed BD patients.
2. To investigate if there is significantly greater comorbidity between BD-I and PTSD than between BD-II and PTSD.
3. To investigate if the presentation of trauma symptoms, in terms of re-experiencing, avoidance, arousal, or dissociative symptoms, is the same across BD-I and BD-II.

Our secondary research objectives were:

1. To investigate if a lifetime PTSD diagnosis as compared to never having received a PTSD diagnosis is associated with a history of psychotic symptoms, suicidal ideation and suicide attempts, current rapid cycling, an earlier onset of disease, a lower level of functioning, and a greater degree of cognitive impairment.
2. To investigate if reporting having experienced sexual abuse, compared to not reporting having experienced sexual, is associated with a history of psychotic symptoms, suicidal ideation and suicide attempts, current rapid cycling, an earlier onset of disease, and a greater number of hospital admissions.
3. To investigate if reporting having experienced physical abuse, compared to not reporting having experienced physical abuse, is associated with a history of psychotic symptoms, suicidal ideation and suicide attempts, current rapid cycling, an earlier onset of disease, and a greater number of hospital admissions.

## Materials and methods

### Data

The data in this paper is the baseline data from a study evaluating the effectiveness of a trauma-focused therapy in traumatized bipolar patients (23). This was a multicenter project comprising three hospitals from the Barcelona area of Spain (Hospital Benito Menni, Hospital Clínic of Barcelona and Hospital Parc de Salut Mar). The trial was registered prior to starting enrolment at Clinical Trials (https://clinicaltrials.gov) under reference NCT02634372.

### Participants

Participants who met criteria for BD-I or BD-II according to DSM-IV criteria, based on clinical interview and a review of case notes, were referred to the study by their referent psychiatrist. The inclusion criteria for participants was: (1) to be aged between 18 and 65; (2) to have experienced two to six affective episodes over the previous 12 months; (3) current clinical status of euthymia or subsyndromal symptoms at the moment of the assessment, defined by a score representing the past week on the Bipolar Depression Rating Scale (BDRS) of <14 and a score representing the previous 2 days on the Young Mania Rating Scale (YMRS) of <12; (4) Presence of a traumatic event according to the Clinician Administered PTSD CAPS-DX scale 0; (5) Current trauma symptoms as indicated by a score >0 on the Impact of Events Scale-Revised (IES-R). The inclusion criteria were designed to enable the testing of the primary hypothesis of the clinical trial comparing EMDR therapy with Supportive Psychotherapy. The EMDR therapy protocol was designed for use with all patients excluding those with active acute symptoms in the present moment, and therefore we screened for active acute symptoms. To be able to test the impact on number of affective episodes, it was necessary to include patients with multiple previous episodes, and the 12-month criteria ensures recent instability as well as permitting classification into rapid cycling (≥4 episodes in the previous year) or not. Exclusion criteria were: (1) current substance abuse/dependency, i.e., not meeting criteria for early remission (three to 12 months without meeting criteria) or sustained remission (over 12 months without meeting criteria) (25), with the exception of nicotine; (2) neurological disease or brain trauma history; (3) current suicidal ideation; (4) having received a trauma-focused therapy within the previous 2 years.

### Variables of study

Patient data was collected by trained evaluators who were all qualified psychologists or psychiatrists working within the participating centers. Each patient was assigned a code, and this was used throughout the data collection to ensure anonymity. A Case Report Form (CRF) was designed to capture baseline data such as sociodemographic variables and clinical variables related to the onset and course of BD. The following data was collected through clinical interview contrasted with a review of medical case notes:

1. Age of onset, defined as the first manic, hypomanic, mixed or depressive episode as per DSM-IV criteria. This data was based on patient recall during the clinical interview, contrasted with notes from medical records.
2. History of psychotic symptoms, defined as having ever experienced psychotic symptoms in line with DSM-IV criteria, based on clinical interview and a review of medical records.
3. Number of relapses over the last year, with a relapse defined as a manic, hypomanic, mixed, or depressive affective episode as per DSM-IV criteria, with data gathered through clinical interview and a review of medical records.
4. Current rapid cycling, defined as four or more affective episodes over the previous 12 month period, with data gathered through clinical interview and a review of medical records.
5. History of suicide attempts, based on patient recall and review of medical records.
6. Current pharmacological treatment, based on their current prescription, and current psychological treatment, based on patient report and medical records.
7. Family history of psychiatric disorder, based on patient recall and review of medical records.
8. Use of substances, collected through patient self-report.

Clinical features, trauma history and symptomatology, functioning and cognitive impairment were all assessed by means of validated scales. Where available, we used scales specifically designed for use in a BD population, and where this was not possible we used the gold standard or most widely used scale. Clinical severity was measured using the following scales:

1. The Bipolar Depression Rating Scale (BDRS) (26), Spanish validation (BDRS-S) (27). This clinician-administered scale is used to assess depressive and mixed symptoms in BD-I and BD-II patients. The BDRS includes 20 items, which sum to a total score between zero and 50. Scores of <8 indicate euthymia and ≥8 and <14 the presence of subsyndromal symptoms. A score of ≥14 indicates the presence of an acute depressive episode. The Spanish validation was carried out with a relatively small sample size but shows robust psychometric properties and captures depressive and mixed symptoms in Spanish bipolar patients.
2. The Young Mania Rating Scale (YMRS) (28), Spanish validation (29), is a clinician-administered scale composed of 11 items aimed at quantifying the severity of manic and hypomanic features. Of the 11 items, four items (irritability, speech, thought content and disruptive/aggressive behavior) are graded on a scale of 0 to 8, while the remaining seven items are graded on a 0 to 4 scale. Total scores range between 0 and 60: scores of <6 indicate euthymia, between ≥7 and <12 indicate the presence of subsyndromal symptoms, while scores of ≥12 indicate the presence of moderate to severe manic symptomatology. The Spanish validation shows this is a reliable tool for the assessment of manic symptoms in patient with manic or hypomanic symptoms in Spain.

Reported trauma history and symptomatology were evaluated using the below scales:

1. The Clinician-Administered PTSD Scale (CAPS-DX) (30), Spanish validation (31). The CAPS is the gold standard for determining a diagnosis of PTSD according to DSM-IV criteria. It provides a diagnosis of both current and lifetime PTSD. The Spanish validation showed good reliability, internal consistency and rest-retest values, similar to the original version.
2. The Impact of Event Scale-Revised (IES-R) (32), Spanish validation (33). The IES-R is a 22-item self-report scale. It measures the presence of subjective distress related to a specific traumatic event, yielding an overall score and one for each of its three subscales, intrusion, avoidance, and hyperarousal, which correspond to the DSM-IV diagnostic criteria for PTSD. Higher scores indicate greater distress and a score of >32 has been suggested as the cut off for the presence of PTSD symptoms (34). The Spanish validation in a large sample showed adequate internal consistency and convergent validity with other scales of psychopathology, but some difficulties with the test-retest validity.
3. The Holmes-Rahe Life Stress Inventory (35), Spanish validation (36). This scale measures the number of stressful events that have occurred over the previous 12 months. Each potential stressful event is accorded a weighted score depending on how stressful it is estimated to be, and these are summed to provide a total score. A score of under 150 reflects low levels of stress and a low risk of stress-related illness, scores from 150–299 reflect a moderate level of stress which can imply a 50% risk of developing a stress-related illness, and scores of 300 and over reflect a high level of stress which can imply an 80% risk of developing a stress-related illness. The Spanish validation includes a cultural adaptation of the items.
4. Dissociative Experiences Scale (DES) (37), Spanish validation (38). This scale assesses the presence of dissociation, by asking participants the percentage of time they experience a range of dissociative symptoms. The results yield an average total score and an average score for each of the three subscales: amnesia, absorption, and depersonalization. Total scores of 30 or higher indicate a potential Dissociative Identity Disorder. The DES is the scale most often employed to measure dissociative symptoms in bipolar patients (39). The Spanish validation was carried out in healthy adults and in inpatients with schizophrenia. The scale was shown to be valid in both populations, with improved validity in a psychiatric population when the scale was administered by a clinician.

The level of functioning and cognitive impairment was evaluated using the following scales:

1. Functioning Assessment Short Test (FAST) (40). This scale evaluates level of functioning through 24 items assessing six domains: autonomy, work, cognitive functioning, finances, interpersonal relationships, and leisure. Each item is scored from 0–3, and overall scores range from 0-72. Higher scores indicate a lower level of functioning. This scale was originally developed in Spanish for a Spanish population.
2. Screen for Cognitive Impairment in Psychiatry (SCIP) (41), Spanish validation (42). The SCIP is a clinician-administered scale which assesses cognitive impairment in psychiatric patients. This scale briefly assesses five different cognitive domains: immediate verbal learning, delayed verbal learning, working memory, verbal language, and processing speed. The scale provides a score for each subdomain and then a global score obtained by summing all scores. Lower scores indicate a greater level of cognitive impairment. The Spanish validation was carried out in a psychiatric population of patients with schizophrenia, and showed good validity and reliability.

### Data analysis

Since the data in this paper come from a study where the sample size was determined to understand the effectiveness of EMDR therapy compared to Supportive Therapy, in which the sample size calculation was based on a survival analysis using the statistical package “powerSurvEpi” for R (http://www.r-project.org/) (40), we performed a second sample size calculation to ensure the sample size is sufficient to meet the objectives of the current paper. In this case, the sample size was based on a correlation test, given the difficulty of exact data regarding the prevalence of BD patients with a history of psychological trauma in the population. A sample of 79 patients, with a statistical power of 80% and a type I error rate of 0.05, is sufficient to detect low correlations (*R* = 0.31) (43, 44).

All statistical analyses were carried out using STATA Statistics/Data analysis, version 16.1 (StataCorp LLC, Texas, USA). Fitness to parametric assumptions was checked for all variables, and the Shapiro–Wilk test was used to assess the normality of data distribution. With regards to the descriptive analysis of the sociodemographic and clinical data, the arithmetic mean was used for quantitative variables and the proportion for categorical variables. In the case of the impossibility of reconciling patient recall data with medical history, or failure to log a valid response to an item, listwise deletion was applied and analyses are based on the total number of valid responses for each question. The standard area and the confidence interval, set at 95%, were calculated for both quantitative and categorical variables. Pearson Chi squared test was used to analyze the relationship between two binomial categorical variables, and a two-sample *t*-test was used for analysing the relationship with the quantitative dependent variables.

Firstly, the relationship between BD-I or BD-II and a lifetime diagnosis of PTSD was analyzed. The lifetime PTSD group comprised both those with a current and lifetime diagnosis of PTSD; while those who had never met criteria for PTSD comprised the non-PTSD group. The current and lifetime PTSD diagnoses were included together as most of the variables against which it was planned to be analyzed were not current (e.g., lifetime number of hospital admissions, lifetime number of suicide attempts, ever having experienced psychotic symptoms, ever having experienced suicidal ideation). Secondly, analyses were carried out for the relationship between BD-I and BD-II and the impact of the traumatic event (IES-R) and dissociative symptoms (DES). Thirdly, the relationship between a lifetime diagnosis of PTSD and a range of clinical symptoms: having experienced suicidal ideation, attempted suicide, experienced psychotic symptoms, rapid cycling, number of suicide attempts, age of BD onset, number of hospital admissions, stressful events over the previous 12 months (Holmes & Rahe scale), level of functioning (FAST), and level of cognitive impairment (S-SCIP) was analyzed. Fourthly, analyses were carried out to determine the relationship between sexual abuse and four categorical variables (whether the participant had experienced suicidal ideation, had a history of suicide attempts, had experienced psychotic symptoms, and rapid cycling), and two quantitative variables (age of onset of BD, and number of hospital admissions). Finally, this analysis was repeated with physical abuse instead of sexual abuse as the independent variable. In all analyses, *p-*value significance was set at <0.05.

We also present adjusted *p*-values based on applying the Holm-Bonferroni correction for multiple comparisons (45). Although only two independent groups are compared in the study, several dependent variables are evaluated. Performing multiple comparisons of the two independent groups for the independent variables may increase the type I error (alpha α1 type error), increasing the risk of falsely rejecting the null hypothesis, being true in the population. To account for this possible effect, we performed the Holm-Bonferroni procedure. However, applying the adjustment for multiple comparisons increases the risk of a type II error, of falsely accepting the null hypothesis, and this risk is arguably greater where the analyses are pre-planned and based on prior evidence (46–49). Therefore, we present both adjusted and unadjusted *p*-values.

### Ethical approval

The study received ethical approval from the Ethics Committee of the Germanes Hospitalàries del Sagrat Cor de Jesús (reference number: PR-2014-15), the Hospital Clínic of Barcelona (reference number: HCB/2015/1005) and the Hospital Parc de Salut Mar (reference number: 2015/6502/l). All participants signed informed consent prior to enrolment.

## Results

### Sociodemographic and clinical variables

In total, 82 subjects agreed to participate in the study, but three did not complete the baseline assessment, leaving a total sample of 79. Most of our sample were females (77.2%, *n* = 61) and Caucasian (94.6%, *n* = 70). The mean age was 46.56 [SD (standard deviation) ± 8.408] and participants had spent an average of 13.74 (SD ± 3.834) years in education. Of the sample, 40.5% (*n* = 32) were single, 38.0% (*n* = 30) married or in a civil partnership, 1.3% (*n* = 1) widowed and 20.3% (*n* = 16) separated. The majority (61.8%, *n* = 47) were on temporary or permanent sick leave, while 22.3% (*n* = 17) were employed and working either full- or part-time. These results can be seen in full in Table 1.

**Table 1:** Sociodemographic characteristics of the sample.

	**Variable**	**Obs/Freq**	Mean /Percentage^*^	**Std. Err**.	**[95% Conf. Interval]**	
Age		79	46.56	1	45	48
Education (years of studies)		57	13.74	1	13	15
Sex	Male	18	22.8%	0.050	0.142	0.34
	Female	61	77.2%	0.050	0.661	0.858
Race	Caucasian	70	94.6%	0.027	0.857	0.979
	Latin American	3	4.1%	0.024	0.013	0.125
	Asian	1	1.4%	0.014	0.002	0.096
Relationship status	Single	32	40.5%	0.058	0.299	0.528
	Married/civil partnership	30	38.0%	0.057	0.261	0.486
	Widowed	1	1.3%	0.014	0.002	0.096
	Separated/divorced	16	20.3%	0.048	0.130	0.324
Employment status	Student	4	5.3%	0.027	0.021	0.143
	Homemaker	1	1.3%	0.014	0.002	0.096
	Employed full-time	14	18.4%	0.047	0.119	0.308
	Employed part-time	3	3.9%	0.020	0.007	0.108
	Temporary sick leave	32	42.1%	0.059	0.325	0.555
	Permanent disability payments due to mental illness	14	18.4%	0.044	0.098	0.277
	Permanent disability payments for other reasons	1	1.3%	0.014	0.002	0.096
	Unemployed	5	6.8%	0.030	0.029	0.160
	Other	2	2.6%	0.014	0.002	0.096

Data are presented as mean or number (%).

Obs/Freq: Number of cases observed/Frequency; Std. Error: Standard Error; Conf.: Confidence.

* Age and education data are presented as means. The rest of the variables are presented as percentages.

The average age of onset of BD in our sample was 29.53 (SD ± 10.840) years old. Age of onset was significantly higher in BD-II patients than BD-I (33.10 years compared to 28.39 years, *p* = 0.044), and in women compared to men (30.89 years compared to 24.65 years, *p* = 0.017).

Patients had experienced on average 3.27 hospital admission during their lifetime, and 2.48 affective episodes in the previous 12 months. In our sample, 73.4% (*n* = 58) had a diagnosis of BD-I compared to 26.6% (*n* = 21) with a diagnosis of BD-II, 13.9% (*n* = 11*)* experienced rapid cycling, and 46.2% (*n* = 36) had experienced psychotic symptoms. Regarding suicidality, 79.7% (*n* = 63) had experienced suicidal ideation and 39.7% (*n* = 31) had attempted suicide. Data regarding severity was available for 29 patients, and in over half of those cases (51.7%; *n* = 15) the attempt had resulted in severe injury. The most common medications taken by our sample were mood stabilizers (94.6%; *n* = 70) and anti-psychotics (75.7%; *n* = 56).

Comparison of the sociodemographic and clinical variables in the current study, as compared to large BD samples from four other studies which are not specifically in a traumatized population (45–48), as well as sociodemographic data for the Barcelona area can be seen in Supplementary Table S1. Our sample had a higher proportion of females and higher age of onset than other studies. The proportion of BD-II patients was, while a minority, greater than in the majority of other studies. Educational level was lower than in other studies and reflected the norms for the Barcelona area. Suicidality was similar to other studies, while a history of psychotic symptoms and current rapid cycling were lower than in most other studies.

### Reported trauma symptoms and profile

The majority (79.7%; *n* = 59) had experienced a relevant stressful life event prior to onset of BD. Over half of our sample (51.9%; *n* = 40) had a lifetime diagnosis of PTSD according to the CAPS. In more than half of these cases, the lifetime diagnosis of PTSD was current, meaning 27.3% of the total sample (*n* = 21) had a current PTSD diagnosis. To carry out the clinical interview for PTSD, using the CAPS scale, participants are asked for the reported traumatic event which most affects them. In nearly half of cases (45.3%; *n* = 34), this was not related to a specific reported traumatic event category. Following this, physical abuse, sexual abuse, and the sudden death of a loved one were all chosen by 12.0% of participants (*n* = 9), followed by violent death (9.3%; *n* = 7), followed by a life-threatening illness (5.3%; *n* = 4) and transport accident (4.0%; *n* = 3). The average age of the participant at the time of the event was 24.18 (SD ± 16.327). However, approximately half of our sample (50.0%; *n* = 38) reported having experienced sexual abuse, while a lower percentage reported having experienced physical abuse (42.1%, *n* = 32). In our sample, over the previous 12 months, participants reported on the Holmes and Rahe scale having experienced an average of 6.72 stressful events each, with an average total score of 214, indicating a moderate level of stress. These results can be seen in Table 2.

**Table 2:** Clinical characteristics of the sample.

	**Variable**	**Obs/Freq**	Mean/Percentage^*^	**Std. Err**.	**(95% Conf. Interval)**	
Age of onset of bipolar disorder		78	29.53	1	28	33
Number of hospital admissions		77	3.27	1	2	5
Number of affective episodes in the last year		79	2.48	0	2	3
Number of suicide attempts		78	0.85	0	0	1
BDRS (total score)		79	9.29	1	8	11
YMRS (total score)		79	2.23	0	2	3
Holmes and Rahe Scale (number of events)		74	6.72	1	6	8
Holmes and Rahe Scale (total score)		74	214	18	177	251
Age of CAPS event		57	24.18	2	20	29
IES-R (total score)		75	38.95	2.973	33.1	44.8
DES (total score)		74	13.24	1.105	11.1	15.4
BD subtype	BD-I	58	73.4%	0.093	0.447	0.815
	BD-II	21	26.6%	0.093	0.185	0.553
Presence of rapid cycling	No	68	86.1%	0.071	0.6424	0.944
	Yes	11	13.9%	0.071	0.056	0.358
Ever experienced psychotic symptoms	No	42	53.8%	0.093	0.447	0.815
	Yes	36	46.2%	0.093	0.185	0.553
Stressful event at time on onset	No	15	20.3%	0.077	0.079	0.399
	Yes	59	79.7%	0.077	0.601	0.921
Ever experienced suicidal ideation	No	16	20.3%	0.052	0.018	0.275
	Yes	63	79.7%	0.052	0.725	0.982
Suicide attempt	No	47	60.3%	0.056	0.494	0.712
	Yes	31	39.7%	0.056	0.288	0.506
Severity of Suicide attempt	No	14	48.3%	0.098	0.276	0.658
	Yes	15	51.7%	0.098	0.342	0.724
Adherence to treatment	Good	73	94.8%	0.063	0.684	0.964
	Partial	4	5.2%	0.063	0.036	0.316
Mood stabilizers	No	4	5.4%	0.052	0.018	0.275
	Yes	70	94.6%	0.052	0.725	0.982
Antipsychotic	No	18	24.3%	0.071	0.056	0.358
	Yes	56	75.7%	0.071	0.642	0.944
Anxiolytic	No	41	55.4%	0.095	0.214	0.589
	Yes	33	44.6%	0.095	0.411	0.786
Antidepressant	No	42	57.5%	0.097	0.376	0.755
	Yes	31	42.5%	0.095	0.214	0.589
Other Meds	No	59	79.7%	0.077	0.601	0.921
	Yes	15	20.3%	0.077	0.079	0.399
CAPS selected event	Accident (transport)	3	4.0%	0.052	0.018	0.275
	Physical abuse	9	12.0%	0.071	0.056	0.358
	Sexual abuse	9	12.0%	0.063	0.036	0.316
	Life-threatening illness	4	5.3%	0.038	0.005	0.246
	Violent death	7	9.3%	0.052	0.018	0.275
	Sudden death	9	12.0%	0.071	0.056	0.358
	Other	34	45.3%	0.095	0.214	0.589

Data are presented as mean or number (%).

Obs/Freq: Number of cases observed/Frequency; Std. Error: Standard Error; Conf.: Confidence; BDRS: Bipolar Depression Rating Scale; YMRS: Young Mania Rating Scale; IES-R: Impact of Event Scale-Revised; DES: Dissociative Experiences Scale.

* Variables up to and including “DES Score” are presented as means. The rest of the variables are presented as percentages.

Across our sample, the mean score on the IES-R was 38.95, indicating PTSD symptoms. In this scale, participants with a lifetime diagnosis of PTSD had a significantly higher average score of 47.19 compared to 29.72 in the non PTSD group [t(72) = −2.783, *p* = 0.007]. Regarding dissociative symptoms, the average score on the DES was 13.24, and was significantly higher in the PTSD group (an average score of 15.53 compared to 10.75), [t(70) = −2.224, *p* = 0.029].

### BD subtype and presence of lifetime diagnosis of PTSD

The Chi squared analysis found no significant between-group differences between Bipolar Type (BD-I or BD-II) and the presence or not of a lifetime diagnosis of PTSD [χ^2^(1) = 0.702, *p* = 0.402].

### Trauma symptom profile per BD subtype

Our results showed no significant differences in the expression of trauma symptoms between BD subtypes in terms of intrusion, avoidance, hyperarousal, or dissociative symptoms. These results can be seen in Supplementary Table S2.

### Impact of PTSD on disease course and cognition

A lifetime PTSD diagnosis was significantly associated with having experienced psychotic symptoms [χ^2^(1) = 5.404, *p* = 0.02]. Our results showed that PTSD did not have a significant impact on disease course in terms of suicidal ideation or behavior, or rapid cycling (please see Table 3). No significant difference was found between lifetime PTSD diagnosis and age of onset, number of hospital admissions, level of functioning according to the FAST, or cognition according to the SCIP, when compared to the sample without a PTSD diagnosis. These results can be seen in Table 4. The *p*-values were no longer significant following application of the Holm-Bonferroni to adjust for multiple comparisons.

**Table 3:** The impact of a lifetime PTSD diagnosis, sexual and physical abuse on categorical variables of disease course.

		**No**	**Yes**	**Mean**	**Std. Err**.	**95% CI**		**χ^2^(1)**	***P*-value**
**Lifetime PTSD diagnosis**									
History of psychotic symptoms	**No**	24	16	0.333	0.079	0.179	0.487	**5.404**	**0.020**
	**Yes**	12	24	0.6	0.077	0.448	0.752		
History of suicidal ideation	**No**	8	7	0.784	0.068	0.651	0.916	0.208	0.648
	**Yes**	29	33	0.825	0.060	0.707	0.943		
History of suicide attempts	**No**	24	22	0.333	0.079	0.179	0.487	1.079	0.299
	**Yes**	12	18	0.45	0.079	0.296	0.604		
Rapid Cycling	**No**	34	32	0.081	0.045	−0.007	0.169	2.220	0.136
	**Yes**	3	8	0.2	0.063	0.076	0.324		
**Sexual abuse**									
History of psychotic symptoms	**No**	24	17	0.351	0.078	0.198	0.505	3.065	0.080
	**Yes**	13	21	0.553	0.081	0.395	0.711		
History of suicidal ideation	**No**	10	6	0.737	0.071	0.597	0.877	1.267	0.260
	**Yes**	28	32	0.842	0.059	0.726	0.958		
History of suicide attempts	**No**	22	24	0.405	0.081	0.247	0.564	0.108	0.742
	**Yes**	15	14	0.368	0.078	0.215	0.522		
Rapid Cycling	**No**	36	30	0.053	0.036	−0.018	0.124	**4.146**	**0.042**
	**Yes**	2	8	0.211	0.066	0.081	0.340		
**Physical abuse**									
History of psychotic symptoms	**No**	25	16	0.432	0.075	0.285	0.578	0.199	0.656
	**Yes**	19	15	0.484	0.090	0.308	0.660		
History of suicidal ideation	**No**	10	6	0.773	0.063	0.649	0.897	0.176	0.675
	**Yes**	34	26	0.813	0.069	0.677	0.948		
History of suicide attempts	**No**	30	16	0.318	0.070	0.181	0.456	2.105	0.147
	**Yes**	14	15	0.484	0.090	0.308	0.660		
Rapid Cycling	**No**	36	30	0.182	0.058	0.068	0.296	2.308	0.129
	**Yes**	8	2	0.063	0.043	−0.021	0.146		

Std. Error: Standard Error; CI: Confidence Interval.

**Table 4:** Impact of lifetime diagnosis of PTSD on quantitative variables of disease course.

	**Obs**	**Mean**	**Std. Err**.	**Std. Dev**.	**95% Conf. Interval**		***t***	**Deg. Freedom**	***P*-value**
**Age of onset**
No lifetime PTSD Dx	37	27.892	1.809	11.002	23.851	36.788	0.748	74	0.162
Lifetime PTSD Dx	39	31.410	1.716	10.718	27.400	40.275			
**Number of hospital admissions**
No lifetime PTSD Dx	36	2.778	1.105	6.629	0.535	5.021	−0.747	73	0.458
Lifetime PTSD Dx	39	3.744	0.710	4.435	2.306	5.181			
**FAST total**
No lifetime PTSD Dx	37	30.703	2.187	13.304	26.267	35.138	0.748	75	0.457
Lifetime PTSD Dx	40	28.275	2.379	15.045	23.463	33.087			
**SCIP total**
No lifetime PTSD Dx	36	65.667	2.833	16.996	59.916	71.417	−0.237	73	0.813
Lifetime PTSD Dx	39	66.641	2.955	18.455	60.658	72.624			

Obs: Number of cases observed; Std. Error: Standard Error; Conf.: Confidence; Deg.: Degrees of; Dx: Diagnosis; FAST: Functioning Assessment Short Test for Bipolar Disorder; SCIP: The Screen for Cognitive Impairment in Psychiatr.

We conducted sensitivity analyses to understand whether there was a significantly different impact from a current PTSD diagnosis as compared to a historical (but not current) PTSD diagnosis. We found no significant differences between the current and historical PTSD group, or the current and never PTSD groups, on any of the variables tested (total FAST score, total SCIP score, rapid cycling, history of psychotic symptoms, age of onset, history of suicide attempts or suicidal ideation). The only significant difference on these variables when comparing the lifetime PTSD group with the never PTSD group was in the history of psychotic symptoms [χ^2^(1) = 6.175, *p* = 0.013; please see Supplementary Table S3], in line with the findings from our main analysis.

### Impact of sexual and physical abuse on disease course

Sexual abuse was shown to be significantly associated with rapid cycling [χ^2^(1) = 4.15, *p* = 0.042]; results were not significant following application of the Holm-Bonferroni to adjust for multiple comparisons. There was no significant association between sexual abuse and suicidal ideation, psychotic symptoms, or history of ever having attempted suicide, or physical abuse and any of the aforementioned variables (see Table 3). Similarly, there was no significant association between sexual or physical abuse and number of suicide attempts, age of onset of BD, or number of hospital admissions. These results can be seen in full in Supplementary Table S4.

## Discussion

Our study is one of the first to analyze a range of clinical and trauma variables in a sample of BD patients exclusively with a history of psychological trauma. Our sample was mostly Caucasian and 77.2% were female, which was higher than the proportion of females in previous studies with large bipolar samples (see Supplementary Table S1) and despite evidence showing BD is estimated to affect both genders almost equally (49). The large proportion of females in our specific sample of BD patients with a history of trauma may reflect evidence showing women are more likely to experience high-impact trauma, experience trauma at an earlier age, and are approximately two to three times more likely to suffer from PTSD (50). In BD patients, PTSD is a more common comorbidity in female BD patients than in male (49, 51). Just over half of our sample (54.1%) had a lifetime diagnosis of PTSD, with this being current in 30.4% of the total sample, and the average age at which the most important traumatic event occurred was 24.18.

The level of education of our participants was representative for the Barcelona area (see Supplementary Table S2), and lower than in other studies, where the samples were found to be more highly educated than the general population (45, 46, 48). Most patients in our sample were unable to work either temporarily or permanently due to BD. Most of our sample of traumatized BD patients (79.7%) had suffered from suicidal ideation at some point in their lives, and 39.7% had carried out a suicide attempt, comparable to data from other BD samples (see Supplementary Table S2). Of note, current suicidal ideation was a criterion for exclusion in our study. In our sample, 13.9% currently experienced rapid cycling, lower than in other samples but participants were excluded from our study if they had experienced >6 mood episodes in the previous 12 months. A history of psychotic symptoms was present in 46.2% of our overall sample, lower than in some other samples but this may partially be due to the higher proportion of BD-II patients, where psychotic symptoms are not a feature of the disease course.

The average age of participants in our sample was 46.56 years and the average age of BD onset was 29.53 years. The age of onset in our study is much higher than the late teens and early twenties reported in other studies (see Supplementary Table S1). This may partially be explained by the fact that, as compared to other studies, the mean age of our sample was higher, there was a higher percentage of females, and BD-II patients formed a larger proportion of the total sample than in other studies: females were shown in our study to have a significantly later age of onset than males, and the same pattern was found for BD-II patients as compared to BD-I, both patterns reflected in other research (52). However, a surprising finding was that there was no significant association between age of onset and physical abuse, sexual abuse, or a lifetime PTSD diagnosis, although our data showed a non-significant trend towards a higher age of onset than participants who had never had a PTSD diagnosis. This tendency was against expectations, given the prior research showing that childhood trauma is associated with a significantly lower age of onset (12). In our study, in the majority of cases (79.7%), BD onset happened in the context of a stressful life event, which supports previous findings that adverse life events can precede mood symptoms (53, 54), and that traumatic stress disorders can significantly increase the probability of subsequent onset of BD (55). However, our data points to a bidirectional relationship between BD and trauma. Firstly, our results showed that the sample had on average experienced levels of stressful life events in the 12 months prior to evaluation which put them at a 50% risk of developing a stress-related illness, which supports previous findings that BD patients suffer more adverse life events in general than healthy controls (56). Furthermore, the average age for the most traumatic event experienced by our sample was 24.18 (although this figure is subject to bias as this variable was the only one with a substantial amount of missing data [*n* = 57], due to not being systematically collected in the CAPS). In nearly a third of the sample where age of trauma event was available (32.14%, *n* = 18), the traumatic event selected for the CAPS occurred after the onset of bipolar disorder, and in 14.29% (*n* = 8) of cases, the traumatic event stemmed from a BD affective episode, While there is a body of research showing that the experience of psychosis can cause PTSD (57), there has been no similar research to the authors' knowledge into PTSD related to BD mood episodes, despite these clearly having the potential to be traumatic based on our data. Our data points to the importance of research focusing not just on childhood trauma but also on adult traumatic experiences, including experiences related to severe mood episodes and hospitalization experiences, to further elucidate the complex relationship between trauma and clinical disease course. Additionally, clinicians may need to include ongoing assessment of the occurrence and impact of adult trauma experiences, experienced in the context of the BD disease course, in addition to screening for childhood trauma.

The data from our study showed participants experienced on average high levels of current trauma symptoms. The impact caused by the traumatic event, measured by the IES-R, was on average well above the cut off for clinical post-traumatic stress symptoms in the group with lifetime PTSD diagnosis, with an average score of 47.19 compared to a cut off score of >32 (34), indicating high levels of distress caused by the traumatic event. Yet it is of note that the group with no lifetime diagnosis of PTSD also had on average symptoms nearing this cut off point (29.72), suggesting a high level of subsyndromal post-traumatic stress symptoms even in those in our sample without a lifetime PTSD diagnosis. In terms of dissociation, there was a significantly higher level of dissociative symptoms in the lifetime PTSD group than in the non-lifetime PTSD group. However, the scores in both groups were well below the >25 score correlated with PTSD, and in line with the mean score of 14.8 on this scale for BD patients, according to a recent meta-analysis which found bipolar disorder patients to be the psychiatric group with lowest levels of dissociation (58). Our results also support dissociative symptoms not being at a clinical level, even in a sample of traumatized BD patients, and our results indicate that the sample was not characterized by complex PTSD, where it has been argued that dissociation is a major feature (59). Our data is useful given the previous lack of information focusing on dissociative symptoms in severe mental illness (22), but contrasts with some prior studies which have found higher levels of dissociation in BD patients (39, 60). One possible explanation for this is that these studies appear to have applied the DES as a self-administered scale, whereas to improve validity and avoid inflated scores in psychiatric patients, we applied the DES as a clinician-administered scale (38). Additionally, in our study, no participants were in an acute affective phase, and further research can clarify the effect of an acute mood episode on dissociative symptoms.

Of note, in our study, we use retrospective reports of trauma. Retrospective and prospective reports show a poor level of agreement (61), and while retrospective reports of adverse childhood experiences can predict negative life outcomes and psychopathology (62, 63), retrospective reports of childhood maltreatment are more strongly associated with early adult life psychopathology than prospective reports, suggesting that the recollection of having been maltreated is more closely associated with psychopathology than prospective measures (64), although other studies have found both retrospective and prospective reports of childhood trauma predict psychopathology (65, 66). There is a dearth of studies analyzing the association between BD and prospective measures of trauma, and our data should be interpreted in the context of the relationship between patient recall of trauma and clinical BD course.

Regarding our first hypothesis, an association was found between BD comorbidity with PTSD and psychotic symptoms. This no longer reached statistical significance once adjustments for multiple comparisons, to decrease possibility of a type I error (i.e., a false positive) were applied. However, adjustments for multiple comparisons increase the risk of a type II error (i.e., a false negative), and arguably are not indicated in situations where hypotheses are planned a priori based on prior evidence, rather than testing for multiple random associations (67–70). Therefore, we present both adjusted and unadjusted *p*-values and note that the interpretation of results must be made with caution.

The tendency in our results to a link with psychosis is unsurprising given the large body of literature which supports a link between trauma and psychosis generally (71). Psychological trauma may increase risk of psychotic symptoms in people vulnerable to psychosis (72), and previous research has shown a link between comorbid PTSD in BD and psychotic symptoms (73). Within our sample, 46.2% had experienced psychotic symptoms, lower than in some studies which have estimated that over half of BD patients experience psychotic symptoms (74, 75), although our study included BD-II patients. It has been argued that psychotic symptoms do not necessarily reflect a worse disease course as they do not have a significant impact on functioning (75) yet psychotic symptoms can be traumatizing for those experiencing them (76). The association between psychotic symptoms in BD and PTSD and its implications for treatment warrant further research.

No significant association was found between PTSD and an earlier onset of BD or suicidality, which was somewhat unexpected given previous research (6, 7, 51). There was also no significant difference found in level of functioning or cognitive impairment. Our results are striking compared to the wide body of literature showing that a PTSD diagnosis in non-BD patients is associated with increased suicidality (77) and can negatively affect functioning and cognition (78, 79). Furthermore, previous research in BD patients has shown that comorbid PTSD has a significant further negative impact (20) over and above the negative impact that BD itself has on functioning and cognition (80, 81). Our sample only included BD patients with a history of trauma, which is not representative of all BD patients. In fact, a trauma history may be present in as few as 50% of BD patients (82), although other studies suggest higher estimates (73). One possible explanation for our results is the high level of subsyndromal psychological trauma symptoms even in the non-PTSD diagnosis group. Indeed, subsyndromal PTSD has been shown in other populations to be associated with significant psychosocial impairment (83, 84) and with increased suicidality (85). One study which compared three groups of BD patients (patients with comorbid PTSD, patients with trauma but no PTSD diagnosis, and patients with no trauma history), found that BD patients with PTSD were significantly more likely than BD patients with no trauma to have a worse disease course, in terms of significantly more rapid cycling and manic symptoms, but there was no significant difference when comparing the comorbid PTSD group with the trauma group, or the trauma group with the no trauma group (86). The impact of comorbidity between BD and psychological trauma, without a diagnosis of PTSD, warrants further investigation. If psychological trauma without a PTSD diagnosis has a similar impact on disease course, this would have important ramifications for screening for and treating psychological trauma in BD patients.

Our second hypothesis, that there would be significantly more cases of PTSD in BD-I patients than BD-II, was not proven, which contrasted with prior evidence from Hernandez et al. (11). In the study by Hernandez and colleagues, the lifetime PTSD diagnosis was 21.3% in BD-I and 15.6% in BD-II. Unsurprisingly, in our sample of BD patients with a history of trauma, the proportion was higher: 49.1% of BD-I and 57.1% of BD-II patients had a lifetime PTSD diagnosis. It is possible that BD-I patients may suffer more rates of psychological trauma but, within BD subtypes with a trauma history, there is not a significantly greater chance of developing PTSD. Indeed, our third hypothesis, that there would be no difference in trauma symptom profile between BD-I and BD-II was shown to be correct: there was no significant difference in levels of post-traumatic stress symptoms or levels of dissociation. This adds to previous evidence (9, 11) which suggests that the different BD subtypes do not influence the expression of trauma symptoms. This data suggests also that addressing PTSD as a comorbidity in BD does not need to differentiate by BD subtype, which could be a useful insight for planning therapeutic approaches for addressing the presentation of trauma symptoms in BD.

Our fourth hypothesis, that the presence of reported sexual abuse would be correlated with a worse disease course, was proven only in the case of rapid cycling, when *p*-values were unadjusted. This is in line with previous studies that found childhood trauma is related to rapid cycling (12) and sexual abuse is the strongest predictor of it (20). Rapid cycling can indicate poor prognosis and be associated with a greater number of suicide attempts (87). The impact of sexual abuse can be treated with psychological treatments such as Trauma Focused Cognitive Behavioral Therapy (TF-CBT) and EMDR (88), and these therapeutic approaches can be adapted specifically for PTSD within the context of bipolar disorder (89, 90). Further research can investigate whether including trauma-focused treatment for BD patients with a history of sexual abuse can improve symptoms of rapid cycling.

Our fifth hypothesis, that reported physical abuse would be correlated with a worse disease course as compared to not reporting having experienced physical abuse, was not proven with any variables. Our findings for sexual and physical abuse do not support previous findings regarding their negative impact on disease course (6, 14, 20). Our results are the first, to the authors' knowledge, to review the impact of physical and sexual abuse only in a sample of BD patients with a psychological trauma history. Therefore, while sexual and physical abuse have been shown in previous research to have a significant impact on disease course, this effect seems to be muted when compared against people who have suffered trauma but not specifically sexual or physical abuse. Another explanation is that our study did not specify physical and sexual abuse in childhood, and there is a strong body of evidence showing that the impact of trauma is greater in childhood (18). Further research can clarify the specific effects of sexual, physical, and emotional abuse at different life stages within a traumatized sample, and whether these warrant specific treatment approaches.

Regarding the clinical implications of our research, the high rates of PTSD within our traumatized sample reflect an important comorbidity which not only can impact prognosis (13) and treatment outcomes (91) but also warrants treatment as a clinical disorder with its own impact on functioning and suffering. Our research supports not only general screening for comorbid psychological trauma and comorbid PTSD in BD patients, which is already implemented in some countries and settings but is not universal, but also ongoing evaluating of whether there has been a traumatic impact due to a BD mood episode or hospitalization.

Clinicians can use information about comorbid trauma symptoms to tailor the BD treatment plan, paying particular attention to possible indicators of worse prognosis such as rapid cycling or psychotic symptoms. Additionally, the inclusion of pharmacological and psychological treatment for clinical trauma symptoms can help clinicians in alleviating the overall symptomatology in patients, and future research should elucidate if this can improve the prognosis of BD course itself.

Strengths of this multicentric study include the exhaustive trauma evaluation, including dissociative symptoms which have received little attention to date (22), and which were assessed through a clinician-administered scale to reduce bias (38), and the PTSD diagnosis which was determined through clinical interview using the gold-standard CAPS. A further strength is that we included “real world” bipolar patients within a pragmatic randomized controlled trial (RCT) with few exclusion criteria. However, some limitations have to be considered as well. We did not evaluate further psychiatric comorbidities using a (semi) structured diagnostic interview, although comorbidities were checked through a review of case notes. Our patients clinically had further psychiatric and somatic comorbidities and it would have been interesting including this variable in our analysis, as prior results indicate negative effects on the course of the illness (92). Furthermore, our study did not include a control group of BD patients without psychological trauma, as the data was taken from a RCT comparing EMDR vs. ST in trauma-exposed bipolar patients. The lack of a non-traumatized control group makes interpretation of the impact of subsyndromic trauma symptoms more challenging. In our study, we collected data regarding the total lifetime number of BD episodes for each participant but many subjects struggled to identify hypomanic episodes or quantify episodes, so this data was not reliable enough to be used. Additionally, emotional abuse was not evaluated, and the timepoint for reported sexual abuse and physical abuse was not assessed. Due to the cross-sectional design of this study, conclusions about causality cannot be drawn. Trauma history was based on subjective recall, which can result in recall bias (93). However, it has been shown that psychopathology is associated more with subjective than objective recall of traumatic events (94). Additionally, trauma history was gathered through use of the gold standard CAPS interview, and we have clarified throughout the paper that this is reported trauma history.

In summary, our paper provides further evidence of the lack of difference in how trauma symptoms are presented across BD subtypes, and provides important data regarding the high levels of trauma symptoms in BD subjects, even when criteria for a PTSD diagnosis are not met. The evidence shows there are few differences in clinical BD severity between the PTSD and subsyndromic PTSD group, although we also found a possible tendency for there to be a correlation between PTSD and psychotic symptoms, as well as between sexual abuse and rapid cycling, which can be clinically helpful in the identification and treatment of both. It prompts further investigation to understand the impact of comorbidity with a history of psychological trauma in BD patients, including subsyndromal PTSD symptoms, and highlights the importance of screening for psychological trauma in the BD population.

## Data availability statement

The data that support the findings of this study are openly available in Figshare at https://figshare.com/articles/dataset/Bipolar_Disorder_and_psychological_trauma/19601359.

## Ethics statement

The study received Ethical Approval from the Ethics Committee of the Germanes Hospitalàries del Sagrat Cor de Jesús (Reference Number: PR-2014-15), the Hospital Clínic of Barcelona (Reference Number: HCB/2015/1005) and the Hospital Parc de Salut Mar (Reference Number: 2015/6502/l). The patients/participants provided their written informed consent to participate in this study.

## Author contributions

BA conceived the idea for the study and led the study. AM-A coordinated the study. BH, AV-G, IG-S, WL, EJ, MM, LB-P, MR, RC, AM-R, and JC were involved in the recruitment and evaluation of patients and data collection. BH, IG-S, MF-M, and AM-A prepared the data for analysis. DR carried out the statistical analysis. BH worked on the first draft of the paper with BA, AM-A, DR-R, and AV-G. All authors contributed to the interpretation of results and the final draft and approved the final draft.

## Funding

This work was supported by a grant for BA from the Plan Nacional de I + D + i and co-funded by the Instituto de Salud Carlos III-Subdirección General de Evaluación y Fomento de la Investigación, Plan Nacional 2008-2011 with the following Research Project (PI/15/02242). Furthermore, BA has been supported by a NARSARD Independent Investigator Grant from the Brain and Behavior Research Foundation (Number: 24397).

## Conflict of interest

The authors declare that the research was conducted in the absence of any commercial or financial relationships that could be construed as a potential conflict of interest.

## Publisher's note

All claims expressed in this article are solely those of the authors and do not necessarily represent those of their affiliated organizations, or those of the publisher, the editors and the reviewers. Any product that may be evaluated in this article, or claim that may be made by its manufacturer, is not guaranteed or endorsed by the publisher.
