# Supplementary material for: High incidence of PTSD diagnosis and trauma-related symptoms in a trauma exposed bipolar I and II sample
Source: Front Psychiatry. 2022 Oct 20;13:931374. doi: 10.3389/fpsyt.2022.931374 (PMC9632656; doi:10.3389/fpsyt.2022.931374)
Supplement: Supplementary file 1 [file Data_Sheet_1.docx]

**Supplementary Table S1.** Comparison of our sample to other large BD samples on a range of sociodemographic data.

|  |  | **Current study sample** | **BDI-BDC (47)** | **JoBS (2004) (48)** | **STEP-BD (49)^a^** | **Stanley Foundation Bipolar Disorders Network (46)^a^** | **Barcelona population ^b^** |
| --- | --- | --- | --- | --- | --- | --- | --- |
| **Sample *n*** |  | 79 | 217 | 191 | 1000 | 261 | NA |
| **Sample type** |  | BD outpatients with a history of trauma with euthymic or subsyndromic symptoms | Outpatients | In- and out-patients | Any patient presenting for treatment | Outpatients | NA |
| **Diagnosis** |  | BD-I or BD-II as per DSM-IV | BD-I, BD-II, or BD-NOS as per DSM-IV | BDI-I, BD-II, or BD-NOS according to DSM-IV | BDI-I, BD-II, BD-NOS or cyclothymia), diagnostic manual not specified | BDI-I, BD-II, BD-NOS or schizoaffective disorder, bipolar type according to DSM-IV | NA |
|  |  |  |  |  |  |  |  |
| **Country** |  | Spain | Australia | Finland | USA | USA and Netherlands | Spain |
| **Gender** |  |  |  |  |  |  |  |
| ***Female*** |  | 61 (77.2%) | 120 (55.3%) | 101 (52.9%) | 586 (58.6%) | 145 (55.6%) | 52.7% |
| ***Male*** |  | 18 (22.8%) | 97 (44.7%) | 90 (47.1%) | 412 (41.2%) | 116 (44.4%) | 47.3% |
| ***Other*** |  | 0 (0.0%) | 0 (0.0%) | 0 (0.0%) | 2 (0.2%) | 0 (0%) | NA |
| **Age of sample (years) (mean ±SD)** |  | 46.56 ± 8.4 | 38.6 ± 12.6 | 37.7 ± 12.2 | 41.0 ± 12.6 | 43.1 ± 1.3 **^c^** | NA |
| **Ethnicity** |  |  |  |  |  |  | NA |
| ***White or Caucasian*** |  | 70 (94.6%) | NA | NA | 926 (92.6%) | 243 (93.0%) | NA |
| ***Hispanic/Latino*** |  | 3 (4.1%) | NA | NA | 37 (3.7%) | NA | NA |
| ***Black or African American*** |  | 0 (0.0%) | NA | NA | 34 (3.4%) | 4 (2.0%) | NA |
| ***Native American*** |  | 0 (0.0%) | NA | NA | NA | NA | NA |
| ***Asian*** |  | 1 (0.014%) | NA | NA | NA | NA | NA |
| ***Other*** |  | 0 (0.0%) | NA | NA | 2 (0.2%) | 14 (5%) | NA |
| **Relationship status ^d^** |  |  |  |  |  |  |  |
| ***Single*** |  | 32 (40.5%) | 48.0% | 25 (27.8%) | 352 (35.2%) | 80 (30.6%) | 44.8% |
| ***Married*** **^e^** |  | 30 (38.0%) | 35.7% | 38 (42.5%) | 362 (36.2%) | 113 (43.3%) | 41.7% |
|  |  |  |  |  |  |  |  |
| ***Widowed*** |  | 1 (1.3%) | 1.2% | 2 (2.2%) | 16 (1.6%) | 4 (1.5%) | 8.5% |
| ***Separated/divorced*** |  | 16 (20.3%) | 15.2% | 25 (27.8%) | 235 (23.5%) | 64 (24.5%) | 4.9% |
| **Employment Status** |  |  |  |  |  |  |  |
| ***Full time*** |  | 14 (18.4%) | 23.6% | 105 (54.4%) | 345 (34.5%) | 86 (32.9%) | NA |
| ***Part time*** |  | 3 (3.9%) | 15.8% |  | 146 (14.6%) | 35 (13.4%) | NA |
| ***Unemployed*** |  | 5 (6.8%) | 38.2% | 24 (12.6%) | 220 (22.0%) | 17 (6.5%) | NA |
| ***Disabled/medically retired*** |  | 15 (19.7%) |  | 45 (23.0%) | 153 (15.3%) | 55 (21.1%) | NA |
| ***Temporary sick leave*** |  | 32 (42.1%) | NA | NA | NA | NA | NA |
| ***Student*** |  | 4 (5.3%) | 10.4% | 19 (9.9%) | NA | NA | NA |
| ***Retired*** |  | 0 (0.0%) | 3.6% | NA | NA | 13 (5.0%) | NA |
| ***Homemaker*** |  | 1 (1.3%) | 8.5% | NA | NA | 16 (6.1%) | NA |
| ***Other*** |  | 2 (2.6%) | NA | NA | 137 (13.7%) | 39 (14.9%) | NA |
| **Educational level** |  |  |  |  |  |  |  |
| ***Less than high school*** |  | 17 (21.5%) | 20.5% | 75 (39.8%) | 39 (3.9%) | 4 (2.0%) | 22.1% |
| ***High school*** |  | 20 (25.3%) | 13.0% |  | 138 (13.8%) | 11 (5.4%) | 46.4% |
| ***Tertiary education not completed*** |  | 17 (21.5%) | 66.4% | 74 (38.7%) | 823 (82.3%) | 77 (37.9%) |  |
| ***Tertiary education*** |  | 25 (31.6%) |  |  |  | 111 (54.7%) | 30.0% |
| ***Vocational school*** |  | NA | NA | 41 (21.4%) |  | NA | NA |
| **BD subtype** |  |  |  |  |  |  |  |
| ***BD-I*** |  | 58 (73.4%) | 89.7% | 90 (47.1%) | 710 (71.0%) | 211 (80.8%) | NA |
| ***BD-II*** |  | 21 (26.6%) | 10.3% | 101 (52.9%) | 239 (23.9%) | 42 (16.1%) | NA |
| ***BD-NOS*** |  | NA | 0.0% | NA | 41 (4.1%) | 5 (1.9%) | NA |
| ***Cyclothymia*** |  | NA | NA | NA | 0 (0.0%) | NA | NA |
| ***Schizoaffective disorder, bipolar type*** |  | NA | NA | NA | 7 (0.7%) | 3 (0.7%) | NA |
| ***Other*** |  | NA | NA | NA | 3 (0.3%) | NA | NA |
| **Age of onset (years)** |  | 29.5 ± 10.8 | 21.0 ± 7.9 | 21.2 (median) | 17.4 ± 8.6 | 22.9 ± 10.4 | NA |
| **Presence of current rapid cycling** |  | 11 (13.9%) | NA | 62 (32.5%) | 200 (20.0%) | NA | NA |
| **History of psychotic symptoms** |  | 36 (46.2%) | 64.8% | 95 (49.7%) | 390 (39.0%) | 155 (59.4%) | NA |
| **History of suicide attempts** |  | 31 (39.7%) | 40.7% | NA | 357 (35.7%) | 75 (28.7%) | NA |

NA: Not available (data not part of study set or not in comparable format).

**^a^** Later papers have been published from these studies with larger datasets, but these papers have been chosen as having the most complete clinical information for preparation with our study.

b Source: <https://ajuntament.barcelona.cat/estadistica/>

**^c^** Data taken from comparison table in Mitchell et al (47).

**^d^** Unable to calculate *n* for relationship status data in the BDI-BDC study

**^e^** Married refers to married/cohabiting in the BDI-BDC, JoBS and Stanley Foundation Bipolar Network studies. In the present study, it refers to married/civil partnership.

**Supplementary Table S2.** Trauma symptoms by Bipolar Type.

|  | | **Obs** | **Mean** | | **Std. Err.** | **Std. Dev.** | **95% Conf. Interval** | | **t** | **Deg. Freedom** | **P value** |
| --- | --- | --- | --- | --- | --- | --- | --- | --- | --- | --- | --- |
|  |  | | | *IES-Intrusion* | | | | | | | |
| **BD-I** | | 55 | 12.655 | | 1.217 | 9.025 | 10.215 | 15.094 | -0.299 | 73 | 0.766 |
| **BD-II** | | 20 | 13.35 | | 1.929 | 8.628 | 9.312 | 17.388 |  |  |  |
|  |  | | | *IES-Avoidance* | | | | | | | |
| **BD-I** | | 55 | 14.345 | | 1.308 | 9.698 | 11.724 | 16.967 | -0.021 | 73 | 0.983 |
| **BD-II** | | 20 | 14.4 | | 2.293 | 10.257 | 9.600 | 19.200 |  |  |  |
|  |  | | | *IES-Hyperarousal* | | | | | | | |
| **BD-I** | | 55 | 10.963 | | 1.351 | 10.017 | 8.256 | 13.672 | -1.173 | 73 | 0.245 |
| **BD-II** | | 20 | 14 | | 2.150 | 9.614 | 9.501 | 18.499 |  |  |  |
|  |  | | | *IES-Total* | | | | | | | |
| **BD-I** | | 55 | 37.964 | | 3.430 | 25.435 | 31.088 | 44.840 | -0.546 | 73 | 0.587 |
| **BD-II** | | 20 | 41.65 | | 6.056 | 27.083 | 28.975 | 54.325 |  |  |  |
|  |  | | | *DES-Amnesia* | | | | | | | |
| **BD-I** | | 55 | 7.873 | | 1.087 | 8.062 | 5.693 | 10.052 | 0.402 | 72 | 0.689 |
| **BD-II** | | 19 | 7 | | 1.930 | 8.413 | 2.945 | 11.055 |  |  |  |
|  |  | | | *DES-Absorption* | | | | | | | |
| **BD-I** | | 55 | 21.255 | | 1.857 | 13.768 | 17.532 | 24.977 | 0.739 | 72 | 0.462 |
| **BD-II** | | 19 | 18.579 | | 3.000 | 13.078 | 12.276 | 24.882 |  |  |  |
|  |  | | | *DES-Depersonalization* | | | | | | | |
| **BD-I** | | 55 | 6.636 | | 1.243 | 9.218 | 4.144 | 9.128 | 0.809 | 72 | 0.421 |
| **BD-II** | | 19 | 4.842 | | 1.098 | 4.787 | 2.535 | 7.150 |  |  |  |
|  |  | | | *DES-Total* | | | | | | | |
| **BD-I** | | 55 | 13.473 | | 1.320 | 9.783 | 10.828 | 16.117 | 0.351 | 72 | 0.726 |
| **BD-II** | | 19 | 12.579 | | 2.032 | 8.859 | 8.309 | 16.849 |  |  |  |

Obs: Number of cases observed; Std. Error: Standard Error; Conf.: Confidence; Deg.: Degrees of

**Supplementary Table S3**. Comparisons of participants with current PTSD, historical (lifetime but not current) PTSD, and who have never had a PTSD diagnosis.

| **Variable** | **Historical PTSD vs current PTSD** | **Historical PTSD vs never PTSD** | **Current PTSD vs never PTSD** |
| --- | --- | --- | --- |
| **History of psychotic symptoms** | *n*=41, χ^2^(1) = 1.735, p=0.420 | *n*=55, χ^2^(1) =6.175, p=0.013 | *n*=56, χ^2^(1) =2.416, p=0.120 |
| **History of suicidal ideation** | *n*=41, χ^2^(1) =0.286, p=0.867 | *n*=56, χ^2^(1) =0.271, p=0.603 | *n*=57, χ^2^(1) = 0.080, p=0.777 |
| **History of suicide attempts** | *n*=41, χ^2^(1) =0.885, p=0.643 | *n*=55, χ^2^(1) =1.038, p=0.308 | *n*=56, χ^2^(1) =0.411, p=0.521 |
| **Rapid cycling** | *n*=41, χ^2^(1) =1.168, p=0.558 | *n*=56, χ^2^(1) =3.399, p=0.065 | *n*=57, χ^2^(1) =0.499, p=0.480 |
| **Age of onset** | *n*=39, p=0.915 | *n*=55, p=0.248 | *n*=58, p=0.263 |
| **No. of hospital admissions** | *n*=39, p=0.234 | *n*=54, p=0.281 | *n*=57, p=0.915 |
| **FAST total** | *n*=40, p=0.552 | *n*=56, p=0.823 | *n*=58, p=0.310 |
| **SCIP total** | *n*=39, p=0.591 | *n*=54, P=0.604 | *n*=57, p=0.912 |

*n*: number of patients

**Supplementary Table S4.** The impact of sexual and physical abuse on quantitative variables of disease course

|  | **Obs** | **Mean** | **Std. Err.** | **Std. Dev.** | **95% Conf. Interval** | | **t** | **Deg. Freedom** | **P value** |
| --- | --- | --- | --- | --- | --- | --- | --- | --- | --- |
| *Age of onset of Bipolar Disorder* | | | | | | | | | |
| **Sexual Abuse No** | 37 | 29.342 | 1.942 | 11.245 | 23.686 | 3.899 | -0.215 | 73 | 0.831 |
| **Sexual Abuse Yes** | 38 | 28.892 | 1.853 | 10.936 | 23.238 | 33.447 |  |  |  |
| **Physical Abuse No** | 43 | 31.093 | 1.776 | 11.649 | 29.461 | 39.661 | 1.355 | 73 | 0.179 |
| **Physical Abuse Yes** | 32 | 27.625 | 1.759 | 9.951 | 26.109 | 36.077 |  |  |  |
| *Number of Hospital Admissions* | | | | | | | | | |
| **Sexual Abuse No** | 36 | 2.722 | 0.511 | 3.067 | 1.685 | 3.760 | -0.559 | 72 | 0.578 |
| **Sexual Abuse Yes** | 38 | 3.421 | 1.117 | 6.880 | 1.160 | 5.682 |  |  |  |
| **Physical Abuse No** | 42 | 2.524 | 0.437 | 2.830 | 1.642 | 3.406 | -1.027 | 72 | 0.308 |
| **Physical Abuse Yes** | 32 | 3.813 | 1.321 | 7.472 | 1.118 | 6.507 |  |  |  |

Obs: Number of cases observed; Std. Error: Standard Error; Conf.: Confidence; Deg.: Degrees of
